# Influence of Perceptual-Motor Calibration on the Perception of Geographical Slope

**DOI:** 10.1177/0301006620918099

**Published:** 2020-04-11

**Authors:** Sally A. Linkenauger, Megan Rose Readman

**Affiliations:** Lancaster University, Lancaster, UK

**Keywords:** hill slant perception, perception/action, perceptual motor calibration, virtual reality

## Abstract

Individuals drastically overestimate geographic slant. Research has suggested this occurs as the amount of energy it would take to ascend the slope modulates the perceived steepness. Numerous studies have provided evidence that alterations in current physiological potential can influence perceptions of geographical slant. However, it is unclear whether these influences are solely due to one’s actual physiological state or whether anticipation of energy expenditure also influences perceived slope. To investigate this, we manipulated anticipated energy expenditure while maintaining actual physiological state by altering the coupling between optic flow and gait. Using virtual reality, we calibrated individuals to either large changes (low anticipated expenditure) or small changes (large anticipated expenditure) in optic flow when walking at the same speed. Following optic flow calibration, individuals estimated slopes of various degrees. The results obtained provide evidence that perceptions of geographical slant are influenced by anticipated energy expenditure.

When we experience the world around us, it is easy to presume that our spatial percepts are based in physical reality, for example, what we see is what is there. However, individuals drastically overestimate geographical slant. For example, Ffordd Pen Llech, Wales, the steepest street in the world boasts a gradient of 37.45% (Suggitt, 2019), a much more conservative inclination than most would estimate. Some researchers suggest that geographical slant is overestimated because rather than our spatial percepts being based on geometric relationships, we ground our spatial percepts with respect to our physiological potential (Proffitt & Linkenauger, 2013). Put simply, the amount of energy it would take to ascend the slope modulates how steep the slope is perceived (Proffitt et al., 1995).

Consider that the steepest slant most individuals can traverse without clambering is roughly around 50° (Giovanelli et al., 2015). If perceived slant is influenced by the amount of energy it would take to ascend the slope, then we may expect that unascendable slants above 50° will be perceived to be close to 90° (Proffitt et al., 1995). According to this theory, the compression at the high end of the scale leads to expansion on the bottom end of the scale which creates higher percepts of steepness of ascendable slopes and larger differences between the perceptions of different ascendable slopes (e.g., 5° and 10° slopes are perceived as 20° and 30° slopes; Proffitt et al., 1995). As the difference in the amount of energy required to traverse a 5° versus a 10° hill is large, the perceived difference in slope more adequately reflects the difference in energy expenditure than the actual slope.

Numerous studies have provided evidence that current physiological state influences perception of geographical slant. For example, out-of-shape, fatigued, encumbered, or elderly individuals perceive hills as being steeper than those who are physically fit, energised, unencumbered, or young (Bhalla & Proffitt, 1999). These studies focus on current physiological states. However, because current physiological state typically influences anticipations of energetic expenditure, it is difficult to tease apart which variable influences perceptions of geographical slant.

Perceptual motor recalibration can be used to influence anticipated energetic expenditure without influencing current physiological state. Optic flow is the rate of movement in the visual array as a consequence of one’s movement (Gibson, 1950). In any environment, individuals learn to anticipate the amount of optic flow they should experience as a consequence of a given amount of walking. This learned relationship, known as perceptual motor coupling (van Andel et al., 2017), modulates individuals’ perceptions of energetic expenditure across a given distance. For example, if one experiences little optic flow following a step, they learn that it takes a lot of energy to not go very far. Conversely, if one experiences a large amount of optic flow following a step, they learn that it takes little energy to go far. Subsequently, by recalibrating the relationship between optic flow and gait, anticipation of energetic expenditure can be altered without altering current physiological state. Applying this principle, we analysed the influence of anticipated energy expenditure on slant perceptions by manipulating whether large or small changes in optic flow were experienced when walking at a constant speed.

Fifteen participants walked on a treadmill at a speed of 1.5 km/h and 5° incline for 10 minutes while experiencing either slow or fast optic flow. Participants viewed a virtual reality (VR), comprising a grass plane with a paved path lined with shrubbery and trees, through an Oculus Rift CV1 head-mounted display (see Figure 1). The ground plane was slanted to 5° so that participants experienced walking on a 5° slope. During the slow optic flow condition, the VR moved past participants at a rate of 1 m/s; during the fast optic flow condition, the VR moved past participants at a rate of 7 m/s.

**Figure 1.**
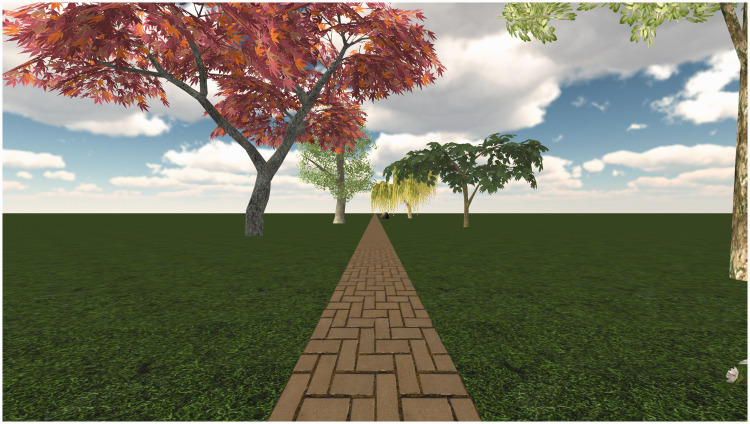
An Example of the Virtual Geographical Hill Slant Display Presented to Participants. *Note:* Please refer to the online version of the article to view the figures in colour.

Following optic flow calibration, participants viewed nine different slopes (0°, 5°, 10°, 15°, 20°, 25°, 30°, 35°, and 40°) from their base twice in random order. Participants verbally estimated the slope of the hill in degrees with 0° being a flat plane and 90° being a vertical cliff. We elected to use verbal estimation as existing research suggests that overall measures of explicit awareness, including both verbal and visual estimations, show the influence of energetics on perception in a similar manner (Proffitt, 2009). Therefore, it would be redundant to include several explicit awareness measures. Furthermore, it has been suggested that due to the necessity for visually guided actions to accommodate to the environment, measures of explicit awareness tend to reflect perception of identification (Proffitt, 2009). However, haptic measurements, such as palm board adjustments, rely on heuristics that bypass the need to represent spatial layout (Fajen, 2007) and, therefore, tend to be a result of perception for action (Creem, & Proffitt, 1998; Proffitt, 2009). On completion of slant estimation in the first optic flow condition, participants repeated the procedure with the other optic flow condition.

Data of one participant were removed from the analysis due to them reporting that they did not understand the slant estimation task. A repeated-measures analysis of variance displayed a significant effect of optic flow with slope estimations being steeper after experiencing slow optic flow (*M *=* *33.57, *SE *=* *2.87) than fast optic flow (*M *=* *30.37, *SE *=* *3.44)—*F*(1, 13) = 6.814, *p *=* *0.022. A significant effect of slope was also found; individuals estimated steeper slopes as being steeper—*F*(1.99, 25.97)= 94.182, *p *<* *.* *001. A significant interaction between slope and optic flow condition was found, in that optic flow calibration influenced estimates of steeper slopes more than shallower slopes—*F*(8, 104) = 2.242, *p *=* *.030 (see Figure 2).

**Figure 2.**
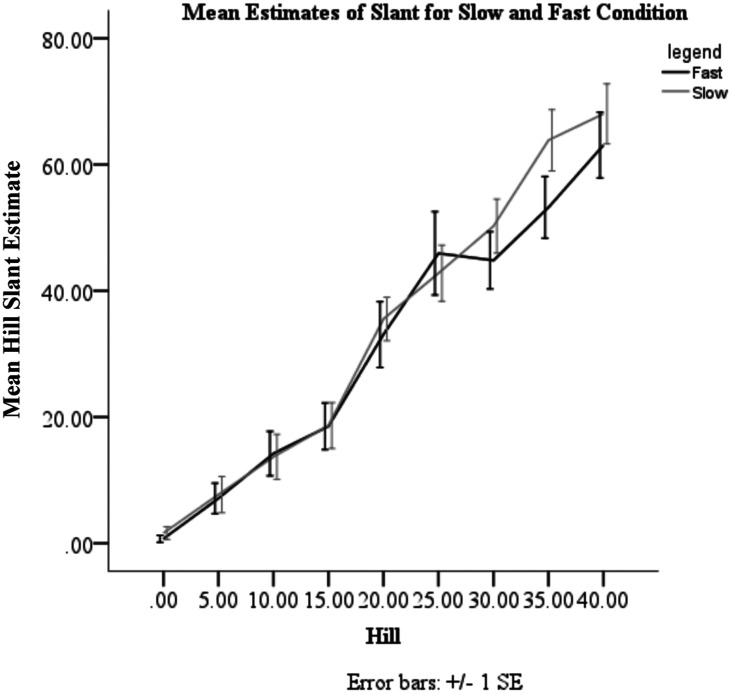
Mean Estimates of Slant for Slow and Fast Condition.

The results suggest that anticipated energetic expenditure does influence perceptions of geographical slant. However, as this study only manipulated anticipated energy expenditure, we cannot suggest that current physiological potential does not influence perceptions of geographical slant. However, it is difficult, near impossible, to manipulate current physiological potential without manipulating anticipated expenditure as well.

Some have suggested that the effect of anticipated energy expenditure is a consequence of response bias (Durgin et al., 2009). However, the interaction effect between slope and optic flow condition provides evidence that optic flow influences estimates of steeper slopes to a greater extent than shallower slopes. If the effect of anticipated energy expenditure was a consequence of response bias, we would expect the influence to be the same regardless of the steepness of the slope being judged. Furthermore, due to the fact that this study does not overtly alter the participant in any way, it will be difficult for the participant to glean the hypothesis and subsequently alter their behaviour in accordance with this knowledge. To conclude, this study provides evidence that perceptions of geographical slant are influenced by anticipated energy expenditure.
